# Effect of a Single Finnish Sauna Session on White Blood Cell Profile and Cortisol Levels in Athletes and Non-Athletes

**DOI:** 10.2478/hukin-2013-0075

**Published:** 2013-12-31

**Authors:** Wanda Pilch, Ilona Pokora, Zbigniew Szyguła, Tomasz Pałka, Paweł Pilch, Tomasz Cisoń, Lesław Malik, Szczepan Wiecha

**Affiliations:** 1 Institute of Biomedical Science, University School of Physical Education, Cracow, Poland.; 2 Department of Physiology, University School of Physical Education, Katowice, Poland.; 3 Rydygier Memorial Hospital, Cracow, Poland.; 4 The State Higher Vocational School, Nowy Sącz, Poland.

**Keywords:** sauna bath, white blood cell profile, cortisol, immunology

## Abstract

The aim of the present study was to investigate the effect of Finnish sauna bathing on a white blood cell profile, cortisol levels and selected physiological indices in athletes and non-athletes. The study evaluated 9 trained middle-distance runners and 9 male non-athletes. The subjects from both groups participated in 15-minute sauna sessions until their core temperature rose by 1.2°C (mean temperature in the sauna room was 96° ± 2°C; relative humidity was 15 ± 3%) with a 2 minute cool down with water at a temperature of 19–20°C. Body mass was measured before and after the session and blood samples were taken for tests. Rectal temperature was monitored at five-minute intervals during the whole session. Serum total protein, haematological indices and cortisol levels were determined. Sauna bathing caused higher body mass loss and plasma volume in the athletes compared to the group of non-athletes. After the sauna session, an increased number of white blood cells, lymphocyte, neutrophil and basophil counts was reported in the white blood cell profile. Higher increments in leukocyte and monocyte after the sauna bathing session were recorded in the group of athletes compared to untrained subjects. The obtained results indicated that sauna bathing stimulated the immune system to a higher degree in the group of athletes compared to the untrained subjects.

## Introduction

Finnish sauna has a substantial effect on the human body. Alternate hot and cold conditions used in sauna bathing are considered to accelerate biomedical athletic recovery and are frequently used as therapies in sport, recreation and rehabilitation. During a sauna session, human body is alternately exposed to hot and cold stimuli. Hot air in the sauna room affects the skin and the respiratory system. Consequently, this leads to a rise in body core temperature up to 39°C, while the temperature at the skin surface might even increase to 42°C (Kauppinen, 1989). If heat release is impossible, the human body is likely to overheat (Hannuksela, 2001; Kukkonen, 2006).

Proper sauna session should be completed with a fast cool-down so that the body is quickly cooled and stops sweating. Prolonged sweat release leads to a decrease in intravascular plasma volume and consequently causes an increase in hematocrit (HCT), total red blood cell (RBC) count and leukocyte (WBC) count (Blum, 2007).

Increased core temperature induced by a sauna session promotes the release of adrenocorticotropic hormone, cortisol and catecholamines (Ježova et al., 1994; Pilch et al., 2007). Increased concentrations of cortisol after a sauna session provide a very sensitive indicator of thermal stress as a response to overheating during sauna bathing (Follenius et al., 1982).

A reduction in hormone levels to the normal status takes more than 24 hours (Pilch at al., 2007).

Several studies have documented the positive effect of sauna on the human body (Kauppinen, 1989; Nguyen et al., 2004; Kihara et al., 2004; Kukkonen, 2006; Hannuksela, 2001; Crinnion, 2011). It is widely believed that sauna bathing contributes to more effective rehabilitation after injuries and brings relief in pain syndromes (Masuda et al., 2005 a, b). Sauna sessions are recommended not only for relaxation and recovery but they also help patients with certain dysfunctions of the respiratory system (Laitinen et al., 1998), chronic rheumatic disorders, degenerative joint diseases and arterial hypertension (Eisalo et al., 1995; Ikeda et al., 2005; Saadat et al., 2009). Finnish sauna sessions promote a response from endocrine glands (Pilch et al., 2007) and stimulate the immune system (Dugué and Leppänen, 1999). It has been documented that hot-cold bathing reduces susceptibility to colds and prevents infections in healthy subjects (Ernst et al., 1990; Dugué et al., 1998).

However, there is little scientific evidence that the repeated exposure to alternate hot-and-cold stress used in Finnish sauna bathing with a body cool-down improves the resistance to infections and infection-related diseases (Dugué and Leppänen, 1999). Changes in the count and activity of white blood cell system observed in subjects who regularly swim in cold water are also very interesting (Dugué et al., 1998).

No studies have been carried out to date on the effect of sauna bathing finished with a cool-down on white blood cell profile in athletes, although the results of numerous studies have found immune system adaptations regardless of the duration and training load (Nieman and Pedersen, 1999; Koch, 2010). These changes concern a variety of components in the immune system, such as leukocyte count and composition, or properties of granulocytes (Koch, 2010). A factor which induces a response of the immune system under these conditions might be the damage to the muscle tissue which consequently leads to an inflammatory response caused by an increase in the number of leukocytes and changes in their composition (Leandro et al., 2007). Furthermore, a study carried out in this area has demonstrated that a prolonged physical exercise might cause immunosuppression (Gleeson, 2007). Therefore, it seemed essential to verify whether sauna bathing has an effect on the immune system and whether the effect of sauna bathing differs between trained and untrained subjects.

The aim of the study was to determine the effect of a Finnish sauna session with a cool-down on the white blood cell profile, cortisol level and selected physiological indices in athletes and non-athletes.

## Material and Methods

### Subjects

The study evaluated two groups of men, who were volunteers aged 21.6 ± 0.52 years (athletes) and 21.7 ± 0.48 years (non-athletes), without any health problems reported in the interview and during a medical examination and without any contraindications for sauna bathing. The control group (n=9) consisted of untrained men (university students) who participated only 2 times a week in physical education classes included in the university curriculum. The study group (n=9) was comprised of middle-distance runners (800/1000/1500 m) with a similar period of training experience (5 ± 1.2 years). The subjects were advised not to consume caffeine, alcohol, dietary supplements, vitamins and not to participate in other sauna sessions for two weeks before and during the experiment.

The experiment was approved by the Bioethics Committee for Clinical Research at the Regional Medical Chamber in Cracow, Poland (No. 202KBL/OIL/2003).

### Research Protocol

The study consisted of passive heating of the subjects’ body in a Finnish sauna room. Mean temperature in the sauna at the height of face was 96° ± 2°C and the mean humidity was 15 ± 3%. The subjects stayed for 15 minutes in a hot sauna chamber in a reclining position. The sauna session was finished with a 2-minute cool-down in the shower with constant water temperature (19–20°C). These activities were repeated until the rectal temperature rose by 1.2°C.

### Measurements

Rectal temperature was monitored during sauna bathing at 5-minute intervals using a MRV-A ELLAB electric thermometer (Denmark) with the accuracy of 0.05°C. Heart rate (HR) was measured with a Polar heart rate monitor, whereas thermal sensations were assessed on the Bredford’s scale.

Body mass was evaluated before and after the sauna bath by means of a Sartorius electronic scale (with the accuracy of 0.001 kg). Based on the changes in body mass, the authors calculated the degree of dehydration.

Furthermore, before and after the sauna bath, blood samples were taken from the cubital vein to three test tubes (the first with EDTA, the second heparynised, the third - native blood) before and 3 minutes after the sauna session.

The morphological blood indices were determined in the EDTA blood with division of leukocytes into 5 fractions: neutrophils, lymphocytes, monocytes, eosinophils, basophils, using Sysmex XE-2001 (Sysmex Corporation).

Serum total protein was evaluated using the biuret method by means of the Hitachi 917 Modular P analyser.

Cortisol levels in blood serum were determined using the electrochemiluminescence method by means of Roche Modular E apparatus (method’s sensitivity: 2μg·l^−1^).

Laboratory determinations of biochemical and hematological indices were carried out in the Department of Clinical Biochemistry, Jagiellonian University Medical College.

Changes in plasma volume were calculated from the changes in serum total protein concentrations determined before and after the experiment, using the formula as follows:

ΔPV = −100 * [(Bk − Bp)/ (Bk)]

Bk: final protein determined after the test
Bp: initial protein determined before the test
ΔPV: changes in blood plasma volume (Harrison et al., 1982).

Due to the changes in plasma volume as a response to dehydration during sauna bathing, cortisol levels after the sauna session were corrected according to Kraemer’s methodology (Kraemer et al., 1989).

$${\text{W}}_{\text{sk}}=(\%\Delta \text{PV}*\;0,01*\;{\text{W}}_{\text{po}})+{\text{W}}_{\text{po}}$$

W_sk_: corrected level
W_po_: level after a sauna bath

### Statistical Analysis

Basic numeral characteristics were determined for the variables studied: arithmetic means and standard deviations. Differences between values obtained after and before sauna bathing were calculated and expressed as deltas (Δ).

Before the significance of differences was evaluated, the authors verified the consistency of the distribution of the variables with normal distribution by means of the Shapiro-Wilk test. If the distribution was not normal, the differences in means were measured with non-parametric tests.

In order to determine statistical significance of the differences between the means the authors used the Wilcoxon signed-rank test for dependent samples (such as: white blood cells count, rectal temperature, changes of plasma volume and body mass) and the Mann-Whitney U test for independent samples (such as anthropometric parameters). Statistical significance between the means was set at p<0.05. The relationships between the indices were evaluated using Pearson’s linear correlation with the level of statistical significance set at p<0.05.

## Results

The group of trained men was characterized by significantly lower fat content and lower body mass (p<0.05) compared to the non-athletes (Table 1). The athletes participating in the study had a training experience of 5±1.2 years and all of them were middle-distance runners who trained 5 times a week. At the time of the study, the athletes were in the period of detraining: their last training session and participation in competition was two months prior to the commencement of the study.

Trained and untrained subject’s body core temperature increased by 1.2 °C after similar duration of the sauna session (Table 2). Before the sauna sessions, the body core temperature in the studied athletes was similar to the temperature recorded for the non-athletes. Mean value of rectal temperature measured in the studied athletes after the sauna sessions did not differ statistically from the mean core temperature of the untrained subjects (non-athletes).

A statistically significant (p<0.05) body mass loss was observed after sauna bathing in both trained and untrained subjects (Table 3). Relative body mass reduction was statistically higher in the athletes compared to untrained subjects.

Sauna bathing caused a statistically higher decrease in blood plasma in the athletes compared to the changes observed in untrained subjects (p<0.05) (Table 4).

A statistically lower white blood cell count was observed in the trained subjects before sauna bathing. The Finnish sauna session affected the white blood cell count in men in both studied groups. The white blood cell count rose significantly in athletes as compared to the group of untrained subjects (Table 5). After the sauna session, a significant increase in neutrophils, lymphocytes, basophils and a significant decrease in eosinophils were observed in athletes.

Furthermore, the sauna session caused significantly elevated blood cortisol levels in men from both studied groups. A statistically significant increase in cortisol levels was observed in the non-athletes (p<0.05) (Table 5). No correlations were found between changes in cortisol levels and changes of white blood cells, neutrophils, lymphocytes, basophils and eosinophils (r = 0.21, p=0.345) after sauna bathing in both groups.

## Discussion

The middle-distance runners are exposed to immunosuppression more than the subjects who exercise moderately. The mechanisms behind immunomodulation induced with physical exercise might be related to metabolic, hormonal and physiological changes caused by intensive muscular work. In trained subjects, the concentrations of catecholamines and growth hormone are elevated. These hormones are responsible for migration of neutrophils to blood due to the exercise-induced stress (Gleeson, 2002). The elevated blood cortisol levels are also observed after intensive workouts. The hormone is likely to sustain the state of lymphopenia and excess neutrophils after prolonged physical exercise (Gleeson, 2002). Physical exercise generates higher levels of reactive forms of oxygen and nitrogen, which might inhibit function of certain cells in the immune system (Moynihan et al., 1998). On the other hand, a higher risk of exposure to pathogens is generated by intensive training regimes. Sauna affects the human body in many ways and, with its thermal stimuli, it affects the functional status of a number of systems in the human body. As a treatment accompanied with hyperthermia, it induces certain disturbances in water/electrolyte equilibrium, changes in metabolic rate of certain energy substrates, activity of the autonomic nervous system, the hormonal system, load in the cardiovascular system and the respiratory system, changes in the profile of morphotic blood components and activity of the immune system (Ježova et al., 1994; Luurila, 1992; Dugué and Leppänen, 1999; Crinnion, 2011).

The peripheral blood cortisol levels in the experiment increased in both groups of men, but the most intensive secretion of this hormone was observed in untrained men compared to the athletes (Δ 5.59±5.89 vs 4.46±4.51 ng·ml^−1^; p<0.05). This suggests that a sauna bath induces higher thermal stress in untrained subjects compared to athletes. The adaptation to thermal stress in sauna is manifested by a lower increase of the cortisol level (Follenius et al., 1982). Findings of different authors concerning changes in cortisol levels induced by hyperthermia in sauna are not uniform. The most of the studies that have investigated this issue demonstrated that cortisol levels were not increased and it even decreased (Pilch et al., 2007). This was observed in particular in the subjects who had never been to sauna before or who exhibited adaptations after several-time exposure to thermal stress in sauna. According to other authors a substantial secretion of cortisol is observed in the subjects who are not adapted to the hot environment, both in men and women (Ježova et al., 1994; Pilch et al., 2007). It should also be noted that, although Follenius et al. (1982) argue that cortisol is a sensitive indicator of thermal stress, the threshold for the rectal temperature over which cortisol secretion increases is 38°C. In our experiment, rectal temperature at the end of the sauna bathing session exceeded this threshold, whereas in the above studies (Kukkonen, 2006) the rectal temperature did not exceed the level of 38°C and an increase in peripheral blood cortisol concentrations was not observed by these researchers.

The exposure to elevated temperature puts a considerable load on the thermoregulatory and circulatory systems. In order to maintain thermal homoeostasis, the body actively responds to the thermoregulatory mechanisms. An efficient way to dissipate excess heat that reaches the human body from the hot environment is perspiration on the body surface. Intensive sweating, however, is associated with losing water from human body, which is manifested with reduced blood volume and blood plasma volume and losing body mass (Pilch et al., 2010).

After a sauna session with a cool-down, body mass loss in athletes was faster than in the untrained subjects and amounted to 1.43% in athletes and 0.9% in the untrained subjects. Higher body mass loss in the athletes is likely to result from intensive perspiration and more efficient transport of heat. However, it also leads to increased dehydration (Pokora, 2006; Pilch et al., 2007). Sweating response starts earlier and is more intensive in trained subjects (Smorawiński and Grucza, 1994; Okazaki et al., 2002).

In this study, a reduction in plasma volume was observed in the men from both groups. The reduction in plasma volume after overheating in the sauna room amounted to 10.2% in athletes, whereas plasma volume was decreased by 7% (p<0.05).

Sauna effect discussed in this study caused elevated white blood cell (WBC) count, chiefly in the trained subjects, whereas the overall number of white cells in the non-athletes remained unchanged (Table 5). The increase in WBC after sauna bathing was statistically higher in athletes compared to the untrained subjects. Similar changes in the white blood cell profile and cortisol levels have been observed in a study by Dugué and Leppänen (1999) after sauna bathing with a cool-down in ice cold water. The authors observed significant differences in WBC count in trained men involved in regular swimming compared to non-swimmers (Dugué and Leppänen, 1999).

In the white blood cell profile, these authors found a statistically significant increase in neutrophils, lymphocytes, basophils after sauna bathing (only in the athletes) (Table 5) and a reduction in the eosinophil count. Similar findings were reported by Ohira et al. (1981) who demonstrated that the total white blood cell count increases after a dehydration caused by passive overheating. They also reported that the number of eosinophils decreases compared with leukocyte count. The above scientists argued that the exogenous heat exposure is likely to have similar effect on the white blood cell profile compared to physical exercise (Ohira et al., 1981).

Lymphocytes are one of leukocyte fractions which perform essential role in the body immune system. The lymphocyte count in our study rose significantly in the group of trained men. One of the subpopulations of lymphocytes is natural killer lymphocytes (NK). Dayanc et al. (2008) found an increase in NK count as a response to the exposure to thermal stress.

Sauna bathing causes insignificant changes in monocyte count in the groups of trained and untrained subjects. However, an increase in monocyte count was significantly higher in the group of athletes. This might suggest a fast mobilization of cells in the first line of immune defence in this group of subjects.

Overheating human body leads to elevated activity of monocytes (Zellner et al., 2008). These cells control biosynthesis of immunoglobulins, have capacity to break down bacteria and help remove dead tissues; they also impact on the activity of fibroblasts and connective tissue cells (angiogenesis) as well as promote secretion of growth factors. Monocytes and neutrophils represent the body’s first line of immune defence against contagious microorganisms and are involved in inflammatory response in post-exercise muscle damage. Physical exercise might promote phagocytic activity of neutrophils and monocytes while glucocorticoids cause neutrophilia, eosinophilia, lymphopenia and have a suppressive effect on NK cells and T lymphocytes (Koch, 2010).

A significant increase in monocyte count was accompanied by a significant increase in the number of neutrophils and eosinophils only in the athletes, which could have been caused by reduced cortisol secretion compared to the untrained subjects.

Based on the results presented in this study, it is difficult to evaluate a role performed by short-term reversible response of the white blood cell profile to thermal stress in sauna. However, the immune system’s response to the stress induced by the heat in a sauna room is undoubtedly stronger in athletes compared to untrained subjects.

## Conclusions

The following conclusions can be drawn based on the results obtained in the present study:

1. Sauna bathing with a body cool-down causes a significant increase in an overall WBC count only in the group of trained men.
2. Sauna bathing considerably elevates neutrophil count, basophil count and lymphocyte count in the blood of trained men.
3. Sauna bathing causes a significantly higher increase in WBC and monocytes in athletes compared to untrained subjects.
4. Changes in the white blood cell profile suggest a faster mobilization of cells in the first line of immune defence in athletes compared to untrained subjects after a sauna bathing session.

Sauna bathing could be recommended for athletes as a means of enhancing immunological defence.

## Figures and Tables

**Table 1:** A general profile of the group of athletes/T/and non-athletes/N/

		Age (years)	Height (cm)	Body mass (kg)	BMI (kg/m^2^)	Fat (%)
Athletes /T/	*χ̄*	21.6	179	67.7^*^	21.16	7.06^*^
	*± SD*	0.52	7.0	5.19	1.16	1.54
Non-athletes /N/	*χ̄*	21.7	176.4	73.43	23.55	10.22
	*± SD*	0.48	5.44	6.98	1.92	2.23

* Significant differences between the groups T/N at the level of p<0.05

**Table 2:** Mean time of sauna bath and differences in rectal temperature in men from the group of athletes (T) and non-athletes (N)

		Total duration of the sauna session (minutes)	Rectal temperature (°C)	
			Before	After
Athletes /T/	*χ̄*	33.8	37.01	38.21
	*± SD*	9.3	0.32	0.32
Non-athletes /N/	*χ̄*	30.6	37.21	38.41
	*± SD*	9.4	0.37	0.36

**Table 3:** Body mass before and after a sauna session in athletes and non-athletes

		Body mass [kg]		
		*χ̄*	*± SD*	%BM
Athletes /T/	Before	68.2	*5.46*	-
	After	67.3	*5.44*	-
	Δ	−0.95	*0.28*	(−)1.43%^*^
Non-athletes /N/	Before	73	*9.34*	-
	After	72.4	*9.3*	-
	Δ	−0.64	*0.44*	(−)0.9%^*^

* Significant differences between the groups T/N at p<0.05

**Table 4:** Changes in plasma volume (PV) in both groups studied after a sauna session

	Δ % PV	
	*χ̄*	*± SD*
Athletes /T/	−10.2^*^	3.29
Non-athletes /N/	−7.0	3.30

**Table 5:** Changes in white blood cell profile and cortisol levels in the men studied

Index	Athletes /T/			Non-athletes /N/		

	Before	After	Δ	Before	After	Δ
WBC [10^3^/μl]	4.60^#^±0.66	5.25^*^±0.82	0.65^#^±0.59	5.24±1.37	5.63±1.10	0.39±0.69
Neutrophils [10^3^/μl]	2.63±0.57	3.16^*^±0.73	0.53±0.63	2.78±0.81	3.12±0.85	0.34±0.49
Lymphocytes [10^3^/μl]	1.36±0.26	1.49^*^±0.21	0.13±0.16	1.82±0.46	1.89±0.34	0.07±0.33
Monocytes [10^3^/μl]	0.40±0.11	0.41±0.11	0.01^#^±0.07	0.46±0.18	0.46±0.15	0.00±0.08
Basophils [10^3^/μl]	0.02±0.01	0.03^*#^±0.02	0.01±0.01	0.02±0.01	0.02±0.01	0.00±0.01
Eosinophils [10^3^/μl]	0.18±0.17	0.16^*^±0.16	−0.03±0.03	0.16±0.07	0.15±0.06	−0.01±0.04
Cortisol (ng·ml^−1^)	10.99±2.02	15.45^*^±3.32	4.46^#^±4.51	13.11±6.77	18.7^*^±4.03	5.59±5.89

* Statistically significant differences at p<0.05 compared to the levels before sauna bathing

# Statistically significant differences at p<0.05 compared to the group N.
